# Multi-temporal UAV multispectral phenotyping and machine learning for modeling cotton defoliation dynamics across diverse accessions

**DOI:** 10.1016/j.plaphe.2026.100295

**Published:** 2026-10-13

**Authors:** Mingze Li, Qisheng Feng, Xiaofei Li, Guoping Wang, Mingwei Du, Sumei Wan, Hongqiang Dong, Mengyan Ren, Zuoren Yang, Lu Feng, Guodong Chen

**Affiliations:** a Key Laboratory of Genetic Improvement and Efficient Production of Specialty Crops in Arid Areas of Southern Xinjiang, College of Agronomy, Tarim University, Alar, Xinjiang, 843300, China; b State Key Laboratory of Cotton Bio-breeding and Integrated Utilization, Institute of Cotton Research of Chinese Academy of Agricultural Sciences, Anyang, Henan, 455000, China; c State Key Laboratory of Herbage Improvement and Grassland Agro-ecosystems, Key Laboratory of Grassland Livestock Industry Innovation, Ministry of Agriculture and Rural Affairs, Engineering Research Center of Grassland Industry, Ministry of Education, College of Pastoral Agriculture Science and Technology, Lanzhou University, Lanzhou, 730000, China

**Keywords:** Cotton defoliation, UAV multispectral imagery, Machine learning, Spectral indices, Temporal phenotyping

## Abstract

Cotton defoliation is critical for efficient mechanical harvesting, yet its temporal progression across diverse germplasm remains difficult to quantify at high throughput. This study developed a multi-temporal unmanned aerial vehicle (UAV)-based machine-learning framework to characterize post-treatment defoliation dynamics across 419 cotton accessions. Multispectral imagery acquired before and after defoliant application was used to derive spectral and texture features, and an Entropy Weight Method (EWM)-based strategy was used for feature ranking and selection. Multiple regression algorithms were evaluated using accession-grouped cross-validation. Spectral indices provided substantially stronger predictive information than texture features, while relative changes from the pre-treatment baseline further improved model performance. Temporal encoding provided a modest but complementary contribution. Among the evaluated algorithms, Random Forest achieved the best overall performance, with an R^2^ of 0.73 and an RMSE of 10.69%. Accession-level trajectory analysis subsequently identified four post-treatment defoliation patterns, with variation primarily reflected in the magnitude and rate of defoliation progression. These results demonstrate the potential of multi-temporal UAV phenotyping for scalable characterization of cotton defoliation dynamics and provide a practical basis for germplasm evaluation and mechanized harvest management.

## 1 Introduction

Cotton (*Gossypium hirsutum* L.) is a globally important natural fiber crop, playing a key role in both the textile industry and agricultural economy [1]. Rising labor shortages and increasing production costs have accelerated the adoption of mechanized harvesting in many cotton-producing regions [2]. Efficient mechanical harvesting requires timely leaf abscission and synchronized boll opening to reduce fiber contamination, improve operational efficiency, and minimize yield losses [3]. Therefore, precise assessment of cotton defoliation is critical for optimizing harvest scheduling and evaluating cultivars under mechanized production systems.

Conventional defoliation assessment relies largely on manual field surveys, which are labor-intensive, subjective, and difficult to scale to large breeding populations or field experiments (Yu et al., 2016). In recent years, unmanned aerial vehicle (UAV)-based remote sensing has emerged as an effective platform for high-throughput crop phenotyping because of its high spatial resolution, operational flexibility, and relatively low cost [4]. UAV platforms equipped with multispectral sensors have been widely applied to estimate crop canopy structure, biomass, leaf area index, nutrient status, and phenological characteristics [5], [6], [7], [8]. These advances provide new opportunities for non-destructive monitoring of cotton defoliation under field conditions.

Among UAV-derived metrics, vegetation indices (VIs) are widely used to quantify crop physiological status, as they are sensitive to chlorophyll content and canopy reflectance changes. Several studies have reported strong correlations between multispectral VIs and cotton defoliation or boll-opening rates [9], [10]. However, individual spectral indices suffer from saturation effects and high collinearity, particularly during late-season senescence when canopy structure and pigment composition change rapidly [11]. To overcome these limitations, texture descriptors and feature-fusion strategies have been introduced to incorporate structural information and improve prediction stability [9]. In parallel, machine learning algorithms have become increasingly popular for extracting nonlinear relationships between remotely sensed features and crop traits. Recent studies further demonstrated that incorporating temporal information can improve the characterization of crop developmental processes and enhance model robustness [12], [13], [14]. In particular, deep learning (DL) approaches have significantly advanced crop phenotyping by automatically extracting complex non-linear spatio-temporal features [15]. Convolutional Neural Networks (CNNs) have proven highly effective in capturing spatial canopy architectures, while Long Short-Term Memory (LSTM) networks excel in modeling sequential dependencies in multi-temporal datasets [16]. Although DL models have been successfully applied to crop yield prediction and growth monitoring, their specific application to modeling the highly dynamic and accession-specific process of cotton defoliation remains limited.

Despite these advances, cotton defoliation remains a complex physiological process rather than a simple chemical response to defoliant application. The progression of leaf senescence and abscission is influenced by accession-specific differences in hormonal regulation, canopy architecture, maturity synchronization, and environmental adaptation [3], [17]. Consequently, cotton accessions often exhibit markedly different responses to identical defoliation treatments. Recent evidence suggests that genetic variation can influence canopy structural dynamics, chlorophyll degradation patterns, and spectral responses throughout the defoliation period [18]. Such heterogeneity is particularly evident in breeding nurseries, where large germplasm collections representing diverse genetic backgrounds are evaluated simultaneously. Under these conditions, models developed from limited genetic materials may not generalize well across populations. However, a critical research gap remains in current high-throughput phenotyping: the lack of multi-temporal modeling approaches capable of characterizing dynamic defoliation trajectories across large and diverse germplasm populations. Most previous studies have evaluated defoliation as a static physiological state at discrete time points [19]. To address this gap, this study developed a multi-temporal UAV-based framework to characterize post-treatment defoliation dynamics across diverse cotton accessions. Specifically, we aimed to: (1) identify UAV-derived spectral and textural features associated with defoliation rate; (2) evaluate the contribution of temporal information to defoliation-rate estimation; and (3) characterize accession-specific post-treatment defoliation trajectories. This study provides a basis for high-throughput phenotyping of cotton defoliation and supports germplasm evaluation and mechanized harvest management.

## 2 Materials and methods

### 2.1 Experimental site and plant materials

The field experiment was conducted during the 2024 growing season at the Anyang Experimental Station of the Institute of Cotton Research, Chinese Academy of Agricultural Sciences, Henan Province, China (36°3′46.8″N, 114°28′48″E; Fig. 1). The site has a warm temperate continental monsoon climate, with a mean annual temperature of 15.8 °C and annual precipitation of approximately 504.6 mm. The soil is classified as sandy loam.

**Fig. 1:**
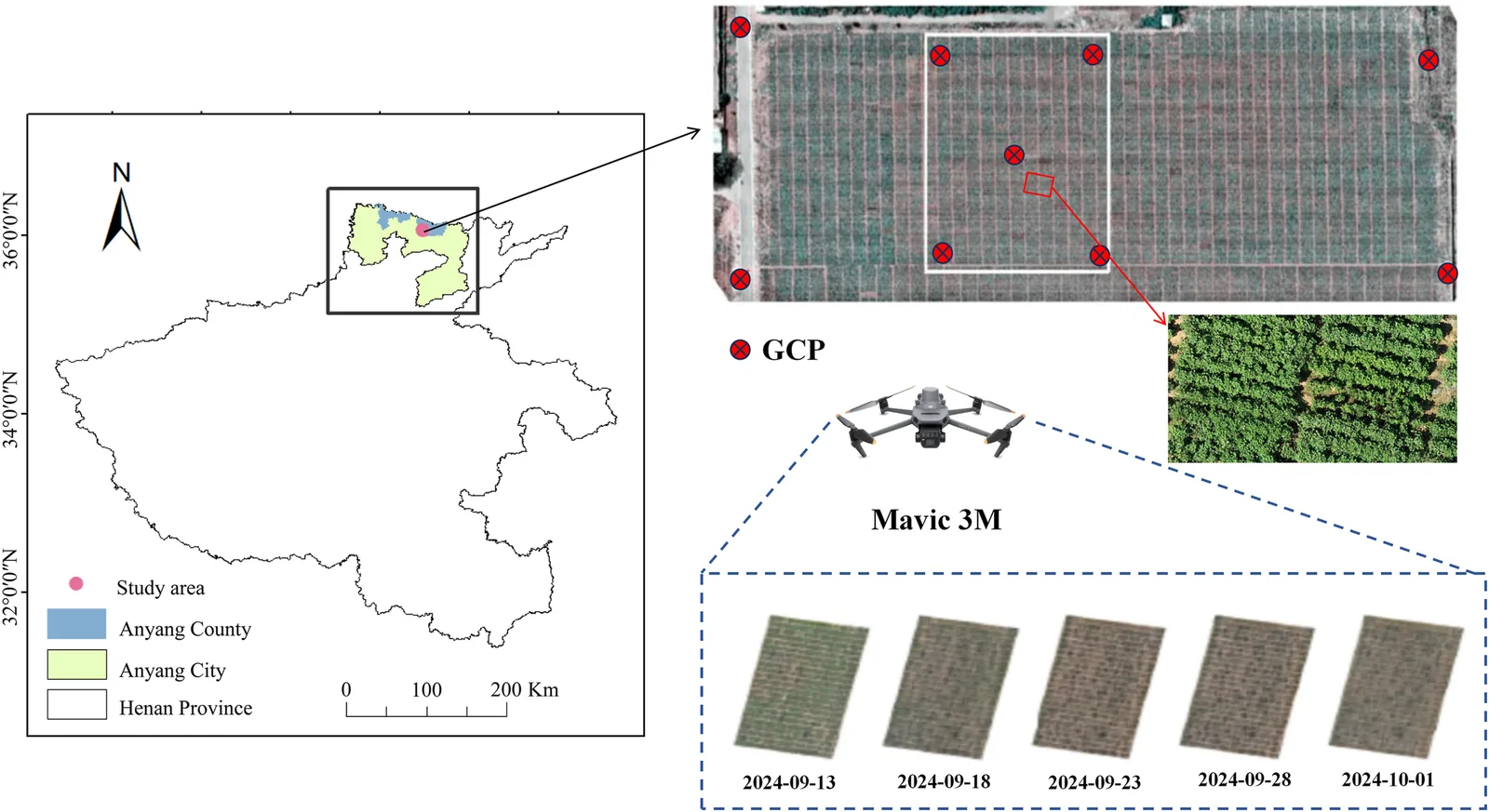
Experimental site, field layout, and UAV monitoring schedule. The study was conducted at the Anyang Experimental Station, Henan Province, China. The experimental field containing 419 cotton accessions is outlined in white. Nine ground control points (GCPs) were established for geometric correction and image georeferencing. Multispectral UAV imagery was acquired immediately before defoliant application and at 5, 10, 15, and 18 days after treatment (Sep 13, 18, 23, 28, and Oct 1, 2024). A zoomed-in inset is provided to illustrate the detailed elongated structure of the individual cotton plots.

A total of 419 upland cotton (*Gossypium hirsutum* L.) accessions representing diverse canopy architectures and geographic origins were evaluated. Based on previously reported population structure and phylogenetic analyses [20], the accessions were assigned to three genetic groups (G1–G3).

### 2.2 Experimental management and defoliant application

The experiment followed a randomized complete block design (RCBD) with two replicate plots per accession. Each plot consisted of two 5-m rows, with 0.8 m between rows and 0.25 m between plants, corresponding to a planting density of approximately 52,500 plants ha^−1^. All plots received the same irrigation, fertilization, and pest and weed management.

Defoliant was applied by UAV on 13 September 2024, when the mean boll-opening rate of the population reached approximately 40%. The mixture contained alkyl ethyl sulfonate (280 g a.i. L^−1^), ethephon (400 g a.i. kg^−1^), and a thiadiazuron–diuron premix containing 360 g a.i. L^−1^ thiadiazuron and 180 g a.i. L^−1^ diuron. Application rates were 600 mL ha^−1^ alkyl ethyl sulfonate, 750 g ha^−1^ ethephon, and 150 mL ha^−1^ thidiazuron–diuron premix, with a total spray volume of 30 L ha^−1^.

### 2.3 UAV data acquisition and image processing

#### 2.3.1 UAV platform and image acquisition

Multispectral UAV imagery was acquired before defoliant application (baseline) and at 5, 10, 15, and 18 days after treatment (DAT). Images were collected using a DJI Mavic 3M equipped with four multispectral bands: green (560 nm), red (650 nm), red-edge (730 nm), and near-infrared (860 nm).

Flights were conducted between 10:00 and 14:00 under stable illumination conditions at an altitude of 25 m, resulting in a ground sampling distance of approximately 1–2 cm. Forward and side overlaps were set to 75% and 80%, respectively.

#### 2.3.2 Image preprocessing and reflectance extraction

Radiometric calibration was performed using calibrated reflectance panels acquired before and after each flight. Nine ground control points (GCPs) surveyed using a high-accuracy GNSS receiver were used for geometric correction. Multispectral images were processed into georeferenced orthomosaics using DJI Terra 5.0.0. Plot-level reflectance values were extracted using an inner-buffer approach to reduce border and inter-plot effects.

### 2.4 Ground measurement of defoliation rate

Ground-based defoliation measurements were conducted concurrently with UAV acquisitions. Because manual assessment of all 419 accessions at each observation date was impractical, stratified proportional sampling was performed according to the three previously defined genetic groups [20].

A total of 60 plots representing 48 unique accessions were selected and monitored throughout the experiment. Twelve accessions were represented by both replicate plots (24 plots), whereas 36 accessions were represented by one plot each. The sampled plots comprised 11 G1, 33 G2, and 16 G3 plots, approximately preserving the population structure of the full germplasm panel. Measurements from the 60 plots at four post-treatment dates yielded 240 plot-date observations for model development and validation.

Within each selected plot, ten central plants were tagged to minimize border effects. Fully expanded leaves were counted before defoliant application (N_0_) and at each subsequent sampling time (N_t_). The defoliation rate (DR) was calculated as:

(1) $$\begin{array}{c} \text{DR}\left(\%\right)=\frac{N_{0}-N_{t}}{N_{0}}\times 100 \end{array}$$

The mean DR of the ten sampled plants was used as the plot-level ground truth.

### 2.5 Feature extraction and selection

Spectral indices (SIs) and gray-level co-occurrence matrix (GLCM)-based texture features were derived from the multispectral imagery (Table 1). The spectral indices were selected to characterize changes in canopy pigment status, reflectance, and structure associated with cotton defoliation [9], [10]. Eight GLCM statistics were used to characterize canopy spatial heterogeneity (Haralick et al., 1973 [21]).

**Table 1:** Summary of spectral indices and GLCM texture features extracted from the UAV multispectral imagery.

Feature Type	Variables	Formula	Reference
Single-band Reflectance	G	Green	
	R	Red	
	RE	Red Edge	
	NIR	Near-Infrared	
Vegetation Indices	DVI	NIR-R	[22]
	CARI	(RE-R)-0.2(RE + R)	[22]
	EVI2	2.5(NIR-R)/(NIR+2.4R + 1)	[22]
	GI	G-R	[22]
	GNDVI	(NIR-G)/(NIR + G)	[22]
	IPVI	NIR/(NIR + R)	[22]
	LCI	(NIR-RE)/(NIR + R)	[22]
	MCARI	[(NIR-R)-0.2(NIR-G)](NIR/R)	[22]
	MDD	(NIR-RE)/(RE-G)	[23]
	MSARI	[RE-R-0.2(RE-G)]RE/R	[23]
	MSR	(NIR/R-1)/√(NIR/R+1)	[22]
	MTCI	(NIR-RE)-(RE + R)	[22]
	NDRE	(NIR-RE)/(NIR + RE)	[24]
	NDREI	(NIR-G)/(RE + G)	[24]
	NDVI	(NIR-R)/(NIR + R)	[25]
	NGBDI	(R-G)/(R + G)	[26]
	NGI	G/(NIR + G + RE)	[26]
	NLI	(NIR^2^-R)/(NIR^2^+R)	[27]
	NRI	(G-R)/(G + R)	[22]
	OSAVI	1.16(NIR-R)/(NIR + R+0.16)	[27]
	RARI	(RE-R)/(RE + R)	[22]
	RDVI	(NIR-R)/√(NIR + R)	[27]
	RESAVI	1.5(NIR-RE)/(NIR + RE+0.5)	[22]
	RVI	NIR/R	[22]
	SAVI2	1.5(NIR-R)/(NIR + R+0.5)	[27]
	TVI	60(NIR-G)-100(R-G)	[22]
	WDRVI2	(0.2NIR-R)/(0.2NIR + R)	[27]
	WDVI	(0.1NIR-R)/(0.1NIR + R)	[27]
Texture Features	GLCM_Mean	Average gray level of the canopy, reflecting overall brightness	[28]
	GLCM_Variance	Dispersion of gray-level values; higher values indicate greater canopy heterogeneity	[28]
	GLCM_Homogeneity	Measures the local spatial similarity and smoothness of the canopy image	[28]
	GLCM_Contrast	Measures local variations; highly sensitive to canopy roughness and deep shadows between leaves	[28]
	GLCM_Dissimilarity	Similar to contrast but scales linearly; measures the distance between neighboring pixel pairs	[28]
	GLCM_Entropy	Measures the randomness and complexity of the canopy structural distribution	[28]
	GLCM_ASM	Angular Second Moment; measures textural uniformity (higher when the canopy surface is highly uniform)	[28]
	GLCM_Correlation	Measures the linear dependency of gray levels of neighboring pixels, reflecting spatial directional patterns	[28]

Note: G, green; R, red; RE, red edge; NIR, near-infrared; GLCM, gray-level co-occurrence matrix; ASM, angular second moment.

GLCM-based texture features were extracted at the plot level, with each experimental plot treated as a single analysis object rather than using a fixed pixel-level moving window. For each plot, an adaptive canopy mask was generated from the NDVI image using Otsu’s thresholding method, which automatically determined the threshold according to the NDVI distribution and excluded non-canopy background pixels from subsequent texture calculation. Image intensities within the masked canopy region were quantized to 32 Gy levels, and GLCMs were calculated using a pixel distance of 1 in four directions (0°, 45°, 90°, and 135°). Texture statistics from the four directions were averaged to obtain direction-independent plot-level values. Eight GLCM statistics were calculated for each multispectral band to characterize canopy spatial heterogeneity.

To reduce feature redundancy, feature importance was evaluated using Pearson correlation, Spearman rank correlation, mutual information, PCA loadings, and Random Forest importance. Scores from the five ranking methods were integrated using the Entropy Weight Method (EWM) [29]. The weight assigned to the j-th ranking method was calculated as:

(2) $$\begin{array}{c} \text{Wj}=\frac{1-e_{j}}{\sum_{j=i}^{m}\left(1-e_{j}\right)} \end{array}$$

where e_j_ is the entropy value of the j-th feature-ranking method and m is the number of methods. Features were ranked according to the integrated scores, and the optimal subset size was determined by incremental feature inclusion based on cross-validation performance.

### 2.6 Dataset construction and temporal encoding

Two feature representations were evaluated: the original post-treatment UAV features and their relative changes from the pre-treatment baseline. For each feature X, the relative change at observation time t was calculated as:

(3) $$\begin{array}{c} \text{RCt}=\left(\text{Xt}-X0\right)/X0 \end{array}$$

where Xt and X0 denote the post-treatment and pre-treatment feature values, respectively.

For each feature representation, models were evaluated using either the full or EWM-selected feature set, with or without DAT as an additional predictor (Fig. 2). DAT was treated as a continuous variable with values of 5, 10, 15, and 18 days. The pre-treatment observations were used only to calculate DR and relative feature changes and were not included as prediction samples.

**Fig. 2:**
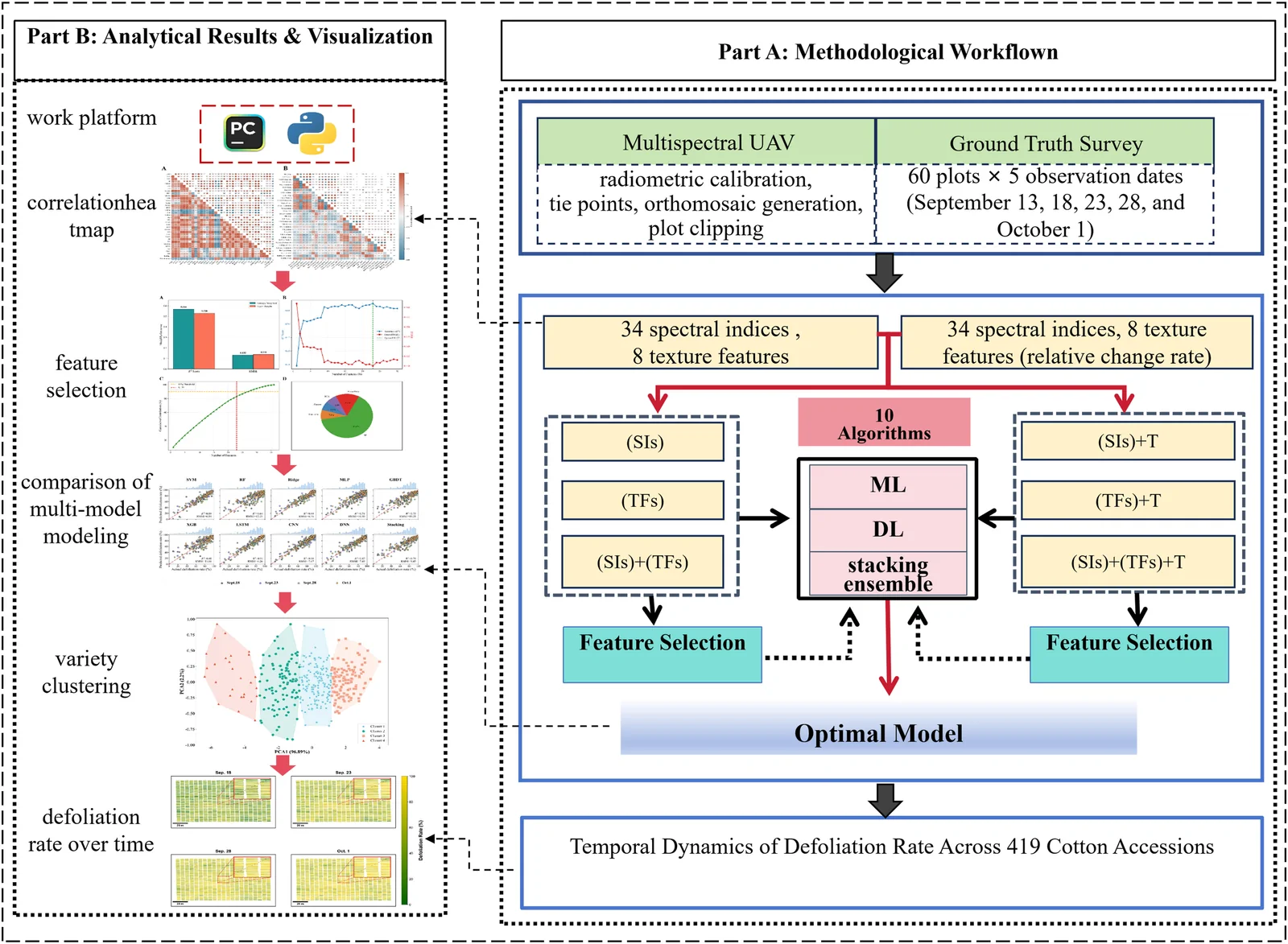
Workflow of the proposed framework for cotton defoliation monitoring and temporal trajectory analysis. Part A illustrates the conceptual data processing pipeline, while Part B displays the corresponding visual outputs and intermediate analytical results generated at each methodological step. Multispectral UAV imagery and ground-based defoliation measurements were used to derive spectral indices (SIs) and texture features (TFs). Feature subsets with and without temporal information (T, days after treatment) were evaluated using machine learning (ML), deep learning (DL), and stacking models. Feature selection was performed to identify the optimal predictor set, and the best-performing model was subsequently used to reconstruct defoliation trajectories and classify accession-specific response patterns.

### 2.7 Model development and evaluation

Ten regression algorithms representing linear, machine-learning, ensemble, and deep-learning approaches were evaluated: Support Vector Machine (SVM), Random Forest (RF), Ridge regression, Multilayer Perceptron (MLP), Gradient Boosting Decision Tree (GBDT), XGBoost, Long Short-Term Memory (LSTM), Convolutional Neural Network (CNN), Deep Neural Network (DNN), and a Stacking ensemble. Model architectures, hyperparameter ranges, and optimization settings are provided in Supplementary Table A1.

All analyses were implemented in Python 3.12 using scikit-learn 1.3.0, XGBoost 2.0.2, and PyTorch 2.1.0. Model performance was evaluated using 10-fold GroupKFold cross-validation with accession ID as the grouping variable. All replicate plots and repeated temporal observations from the same accession were assigned to the same fold, thereby preventing information from the same accession from being shared between the training and validation sets.

Within each cross-validation iteration, all data-dependent preprocessing procedures were performed exclusively using the training data. Specifically, the mean and standard deviation for z-score standardization were estimated from the training fold and subsequently applied to the corresponding validation fold. Model-specific internal optimization or resampling procedures, where applicable, were also restricted to the corresponding training data and did not access the validation fold; detailed settings are provided in Supplementary Table A1. All models were evaluated using the same accession-grouped cross-validation splits for a given data configuration.

Predictive performance was evaluated using the coefficient of determination (R^2^), mean absolute error (MAE), mean squared error (MSE), and root mean squared error (RMSE):

(4) $$\begin{array}{c} R^{2}=1-\frac{\sum {\left(y_{\text{true}}-y_{\text{pred}}\right)}^{2}}{\sum {\left(y_{\text{true}}-{\bar{y}}_{\text{true}}\right)}^{2}} \end{array}$$

(5) $$\begin{array}{c} \text{MAE}=\frac{1}{n}\sum \left|y_{\text{true}}-y_{\text{pred}}\right| \end{array}$$

$$\begin{array}{c} \;\text{MSE}=\frac{1}{n}\sum {\left(y_{\text{true}}-y_{\text{pred}}\right)}^{2}\;\left(6\right) \end{array}$$

(7) $$\begin{array}{c} \text{RMSE}=\sqrt{\text{MSE}} \end{array}$$

where y_true_ and y_pred_ represent the observed and predicted defoliation rates, respectively, ${\bar{y}}_{\text{true}}$ is the mean observed value, and n is the number of samples.

### 2.8 BLUPs derivation and time-series clustering

The optimal model was refitted using the full training dataset with ground-truth observations and subsequently applied to predict plot-level defoliation rates for all 419 accessions at 5, 10, 15, and 18 DAT. To account for spatial field heterogeneity and experimental design effects, accession-level Best Linear Unbiased Predictors (BLUPs) were estimated independently for each observation date using a linear mixed-effects model, with accession and block fitted as random effects. The resulting BLUPs were then utilized as adjusted, accession-level phenotypes for the subsequent trajectory analysis. To ensure equal weighting across all temporal stages during clustering, the four-date BLUP trajectories were z-score standardized across accessions for each observation date independently. K-means clustering, employing k-means++ initialization and Euclidean distance, was then applied to partition the post-treatment trajectory patterns. Candidate cluster numbers ranging from k = 2 to k = 8 were rigorously evaluated using a combination of the sum of squared errors (SSE), Silhouette Score, Calinski–Harabasz index, and Davies–Bouldin index. Principal component analysis (PCA) was employed to project the four-dimensional trajectories into a two-dimensional subspace for visualization. The loadings of PC1 and PC2 were examined to quantify the contribution of each specific observation date to the primary axes of temporal phenotypic variation. Finally, because the BLUPs for the full population were derived from model-predicted rather than directly measured defoliation rates, the resulting trajectories were strictly interpreted as model-assisted phenotypes. This distinction explicitly acknowledges and retains the inherent uncertainty propagated from the first-stage machine learning prediction model.

## 3 Results

### 3.1 Relationship between UAV-derived features and defoliation rate

Correlation analysis revealed clear differences between spectral and texture features in their relationships with defoliation rate (DR) (Fig. 3). Several spectral indices showed strong correlations with DR, whereas most texture features exhibited weaker associations. RARI showed the strongest correlation with DR (r = −0.79, P < 0.001). Other indices, including NDVI, NGBDI, GI, IPVI, WDVI, and WDRVI2, also showed strong correlations with DR, with |r| values reaching 0.76 (P < 0.001; Fig. 3A). Strong correlations among spectral indices further indicated substantial feature redundancy. In contrast, most texture metrics showed relatively weak associations with DR (|r| < 0.30; Fig. 3B), and several features, including red-band Correlation and NIR-band Homogeneity, were not significantly associated with DR.

**Fig. 3:**
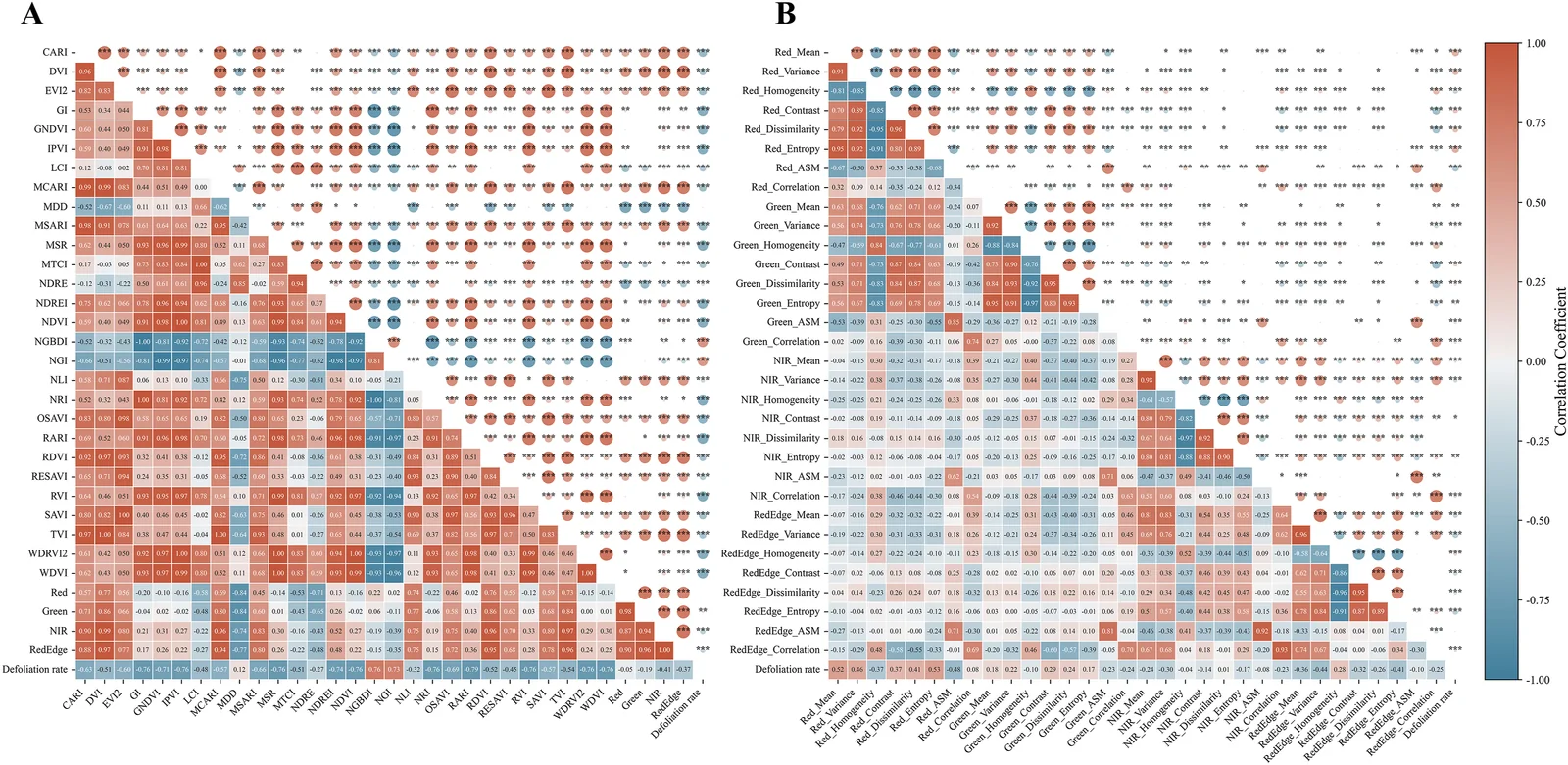
Correlation analysis between UAV-derived features and cotton defoliation rate. (A) Correlation matrix of spectral indices and defoliation rate. (B) Correlation matrix of GLCM-based texture features. Color intensity and ellipse shape indicate the strength and direction of pairwise correlations.

### 3.2 Model performance under different feature representations

Model performance varied substantially across feature representations (Table 2). Using the raw UAV-derived features, spectral indices consistently outperformed texture features. The best model based on raw spectral indices was the MLP, with an R^2^ of 0.65 and an RMSE of 12.14%, whereas the best texture-based model achieved an R^2^ of only 0.46 and an RMSE of 15.21%. Combining spectral and texture features did not improve prediction, with the best combined model reaching an R^2^ of 0.61 and an RMSE of 12.91%. These results indicated that spectral information contributed more strongly to defoliation-rate estimation than texture information.

**Table 2:** Model performance under different feature representations, feature-selection strategies, and temporal configurations for cotton defoliation-rate estimation. SIs, spectral indices; TFs, texture features; DAT, days after treatment.

Data combination	Feature Type	Optimal model	R2	MAE	MSE	RMSE
DAT only	DAT	RF	0.20	13.60	338.14	18.39
All data	SIs	MLP	0.65	9.58	147.36	12.14
	TFs	Ridge	0.45	11.49	231.21	15.21
	SIs + TFs	Ridge	0.61	10.07	166.58	12.91
All data + DAT	SIs	MLP	0.64	9.62	150.35	12.26
	TFs	Ridge	0.52	10.44	201.55	14.20
	SIs + TFs	Ridge	0.62	9.97	161.92	12.73
All data + Feature selection	SIs	LSTM	0.64	9.86	153.35	12.38
	TFs	Ridge	0.48	11.18	218.63	14.79
	SIs + TFs	LSTM	0.61	10.23	163.78	12.80
All data + DAT + Feature selection	SIs	LSTM	0.64	9.82	152.44	12.35
	TFs	MLP	0.56	9.81	187.64	13.70
	SIs + TFs	LSTM	0.64	9.86	154.01	12.41
Relative feature change	SIs	RF	0.71	8.50	122.45	11.07
	TFs	DNN	0.37	12.78	266.78	16.33
	SIs + TFs	RF	0.70	8.84	128.81	11.35
Relative feature change + DAT	SIs	RF	0.72	8.44	119.58	10.94
	TFs	DNN	0.49	10.83	215.31	14.67
	SIs + TFs	RF	0.70	8.81	127.97	11.31
Relative feature change + Feature selection	SIs	CNN	0.70	8.51	124.65	11.17
	TFs	MLP	0.33	12.42	283.32	16.83
	SIs + TFs	RF	0.69	8.93	130.30	11.42
Relative feature change + DAT + Feature selection	SIs	RF	0.73	8.20	114.36	10.69
	TFs	Stacking	0.49	11.07	215.76	14.69
	SIs + TFs	GBDT	0.71	8.43	122.34	11.06

When UAV-derived features were expressed as relative changes from the pre-treatment baseline, spectral indices provided higher predictive performance than their raw representations. The optimal RF model achieved an R^2^ of 0.71 and an RMSE of 11.07%, compared with an R^2^ of 0.65 and an RMSE of 12.14% for the best model using raw spectral indices. In contrast, relative texture features remained weak predictors, and combining spectral and texture changes again provided no additional benefit. These results identified relative changes in spectral indices as the most informative input representation for subsequent analyses.

### 3.3 Effects of temporal integration and feature optimization

#### 3.3.1 Temporal integration

As a temporal-only baseline, the RF model using DAT alone achieved an R^2^ of 0.20 and an RMSE of 18.39%, substantially lower than models incorporating UAV-derived spectral information (Table 2). This result indicates that the pooled predictive performance could not be explained by temporal progression alone.

Adding DAT to the relative spectral-change features produced a modest improvement in RF performance, increasing R^2^ from 0.71 to 0.72 and reducing RMSE from 11.07% to 10.94%. Because the same RF algorithm and spectral-change feature set were used in both configurations, this comparison isolates the incremental contribution of temporal encoding. The improvement was small in absolute magnitude, indicating that most predictive information was already captured by the UAV-derived spectral changes. DAT therefore provided complementary rather than dominant predictive information.

#### 3.3.2 Feature optimization

Feature-importance scores from RF, mutual information, PCA loadings, Pearson correlation, and Spearman correlation were integrated using the EWM. RF importance received the largest weight (68.2%), with the remaining four methods contributing smaller weights to the integrated ranking.

The EWM-based and equal-weight rankings produced nearly identical model performance (R^2^ = 0.682 vs. 0.681; RMSE ≈ 11.1%), suggesting that the weighting scheme had only a minor influence on predictive accuracy. Incremental feature inclusion further showed that model performance stabilized when the top 25 features were included, with little additional improvement beyond this point (Fig. 4B and C; Supplementary Table A2). The top 25 features were therefore retained for subsequent model comparison.

**Fig. 4:**
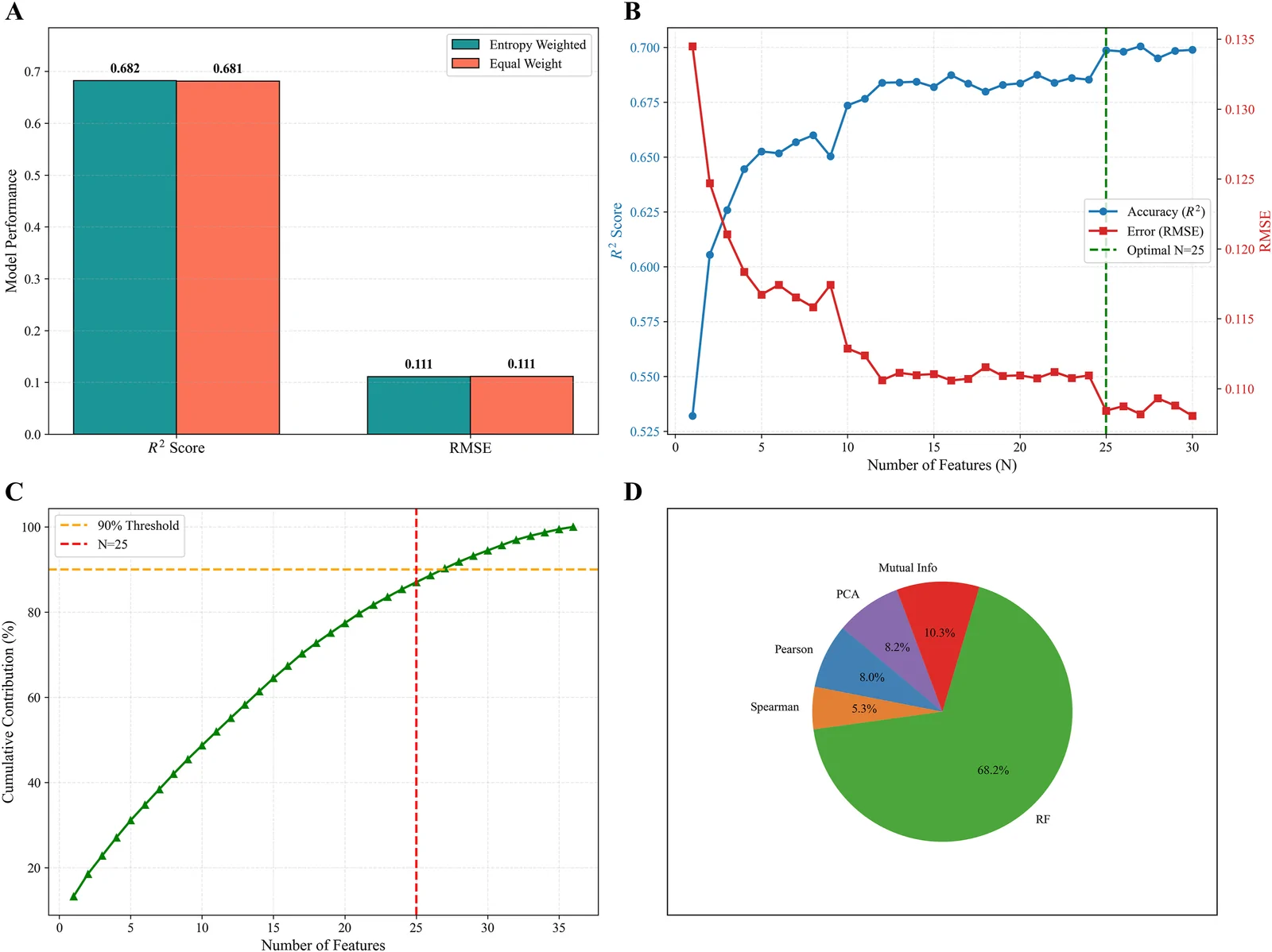
Feature selection based on the entropy weight method (EWM). (A) Comparison of predictive performance between entropy-weighted and equal-weight feature-ranking strategies. (B) Changes in cross-validation performance as the number of selected features increases. (C) Cumulative contribution of ranked features used to determine the optimal subset size. (D) Relative contributions of different feature-ranking methods in the entropy weighting framework.

### 3.4 Optimal model identification and date-specific evaluation

Under the optimal feature configuration, RF achieved the highest overall performance among the ten evaluated algorithms, with an R^2^ of 0.73 and an RMSE of 10.69% (Fig. 5; Table 3). XGBoost and Stacking showed comparable performance, both reaching R^2^ values of approximately 0.72, followed by GBDT (R^2^ = 0.71). SVM, Ridge, MLP, LSTM, and CNN showed intermediate performance, whereas DNN produced the lowest accuracy (R^2^ = 0.59). Overall, tree-based ensemble models achieved the highest predictive accuracy among the evaluated algorithms, whereas the deep-learning models did not outperform them.

**Fig. 5:**
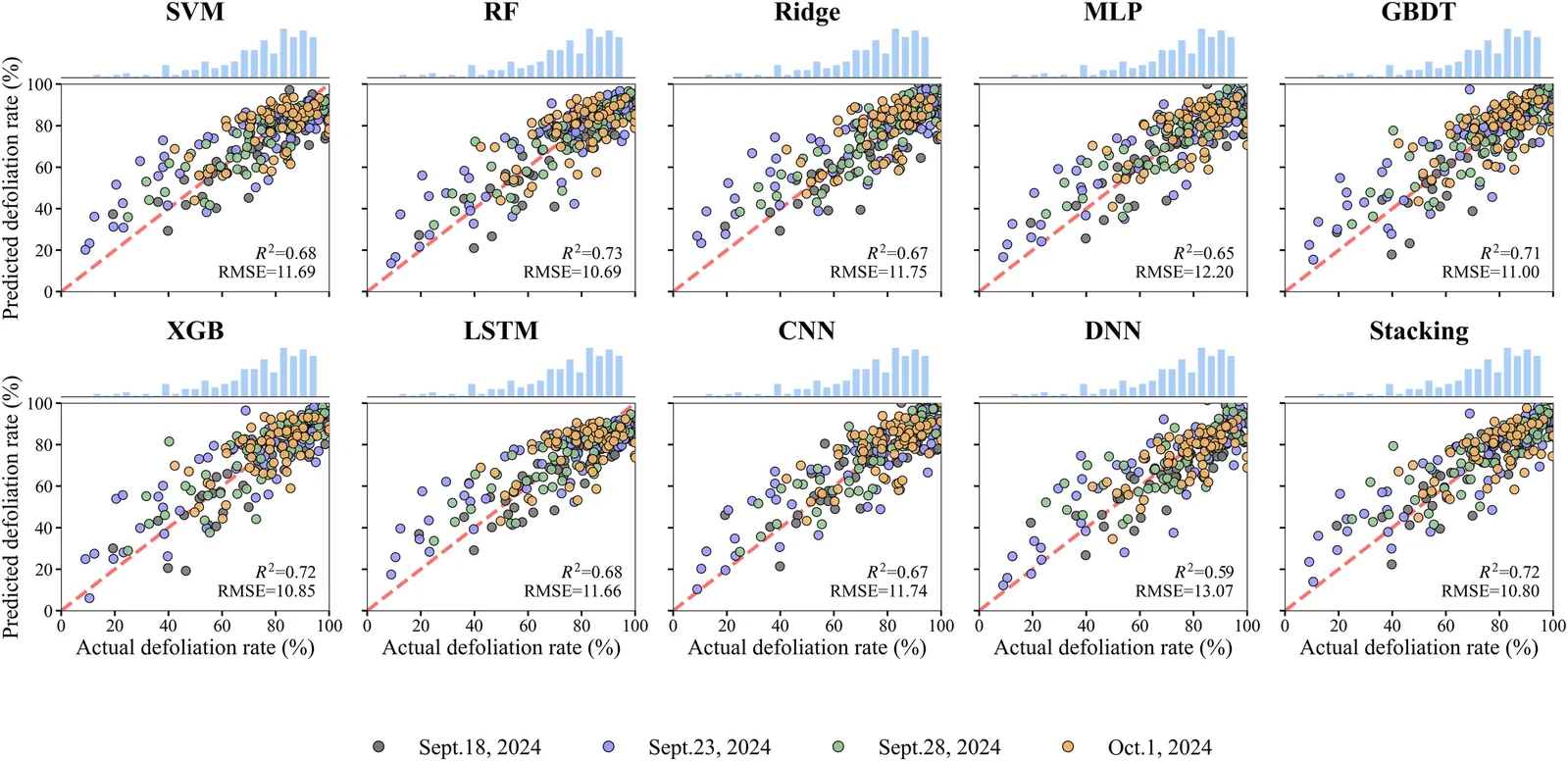
Performance comparison of ten regression models for cotton defoliation-rate estimation using the optimal feature configuration (relative changes in spectral indices combined with DAT and feature selection). Scatter plots show predicted versus observed defoliation rates evaluated using 10-fold accession-grouped cross-validation. Marginal histograms show the distribution of observed defoliation rates. The dashed line represents the 1:1 relationship, and colors indicate observation dates. Model performance is summarized by R^2^ and RMSE.SVM, support vector machine; RF, random forest; Ridge, ridge regression; MLP, multilayer perceptron; GBDT, gradient boosting decision tree; XGB, extreme gradient boosting; LSTM, long short-term memory; CNN, convolutional neural network; DNN, deep neural network; Stacking, stacking ensemble model.

**Table 3:** Performance comparison of ten modeling approaches for cotton defoliation rate estimation.

Model	R^2^	MAE	MSE	RMSE
SVM	0.68	9.36	136.73	11.69
RF	0.73	8.20	114.36	10.69
Ridge	0.67	9.06	138.04	11.75
MLP	0.65	9.22	148.95	12.20
GBDT	0.71	8.23	121.07	11.00
XGB	0.72	8.31	117.79	10.85
LSTM	0.68	9.46	135.99	11.66
CNN	0.67	8.70	137.88	11.74
DNN	0.59	9.54	170.79	13.07
Stacking	0.72	8.11	116.74	10.80

Note: SVM, support vector machine; RF, random forest; Ridge, ridge regression; MLP, multilayer perceptron; GBDT, gradient boosting decision tree; XGBoost, extreme gradient boosting; LSTM, long short-term memory; CNN, convolutional neural network; DNN, deep neural network.

Date-specific evaluation further showed that the RF model retained predictive ability within individual observation dates, with R^2^ values of 0.62, 0.52, 0.50, and 0.57 at 5, 10, 15, and 18 DAT, respectively. Although these values were lower than the pooled R^2^, the positive within-date performance indicated that UAV-derived features captured accession-level variation beyond the overall temporal trend. The final RF model was therefore refitted using the complete ground-truth dataset and applied to the full population to generate defoliation-rate predictions for subsequent trajectory analysis.

### 3.5 Defoliation trajectory clustering and temporal differentiation

The accession-level defoliation trajectories derived from BLUPs were classified into four response groups using k-means clustering. Multiple internal clustering-validation metrics supported k = 4 as a reasonable solution among the candidate cluster numbers evaluated (Supplementary Fig. A1). PCA of the standardized BLUP trajectories showed that PC1 and PC2 explained 87.16% and 9.06% of the total variance, respectively (Fig. 6A). All four observation dates loaded positively and similarly on PC1, indicating that this axis primarily represented overall defoliation magnitude, whereas PC2 captured additional variation in trajectory shape (Supplementary Table A3).

**Fig. 6:**
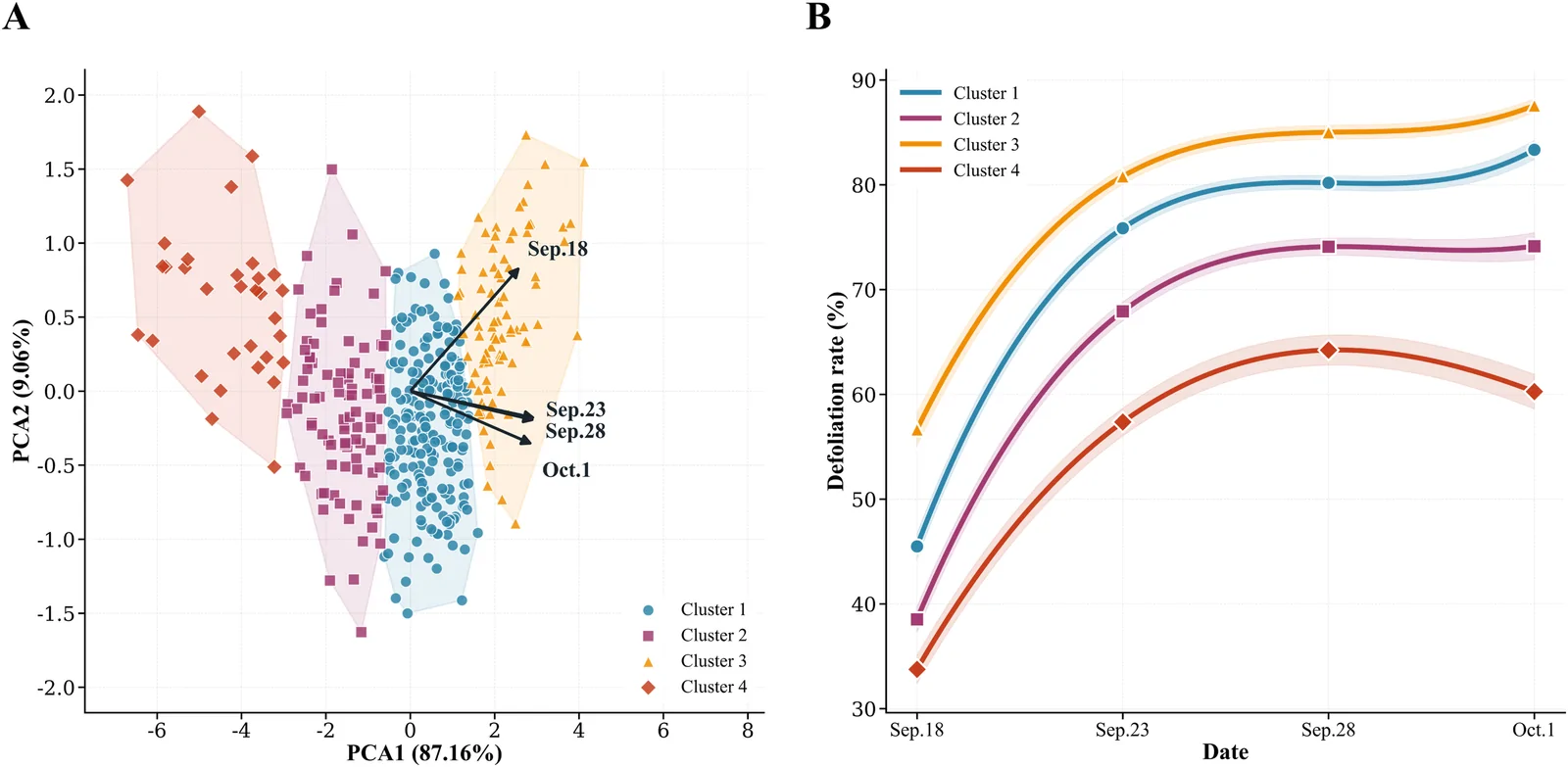
Clustering and temporal trajectories of post-treatment cotton defoliation. (A) PCA biplot based on standardized accession-level BLUPs at 5, 10, 15, and 18 DAT. PC1 and PC2 explained 87.16% and 9.06% of the total variance, respectively. Arrows indicate the loading vectors of the four observations dates, and colored polygons denote k-means cluster boundaries. (B) Mean temporal trajectories of the four retrospectively defined response groups. Shaded bands indicate 95% confidence intervals.

The four groups exhibited distinct temporal patterns (Fig. 6B). Group 3 showed the fastest defoliation progression, increasing from 56.65 ± 5.18% at 5 DAT to 87.57 ± 2.11% at 18 DAT. In contrast, Group 4 showed the slowest progression, increasing from 33.75 ± 4.47% to 60.26 ± 5.48% over the same period. Groups 1 and 2 displayed intermediate trajectories, increasing from 45.49 ± 4.07% to 83.34 ± 3.00% and from 38.52 ± 4.03% to 74.14 ± 4.31%, respectively.

Differences among the retrospectively defined trajectory groups were already apparent at 5 DAT and became more pronounced at subsequent observation dates. Because the 5-DAT observations were included in the clustering analysis, these differences were considered part of the retrospectively defined trajectory patterns rather than an independent early-classification result.

Spatial maps further revealed substantial variation in predicted defoliation rates across plots (Fig. 7). Variation among accessions was visible at 5 DAT, while defoliation increased across most plots at subsequent observation dates. Variation in defoliation progression remained evident among plots throughout the observation period.

**Fig. 7:**
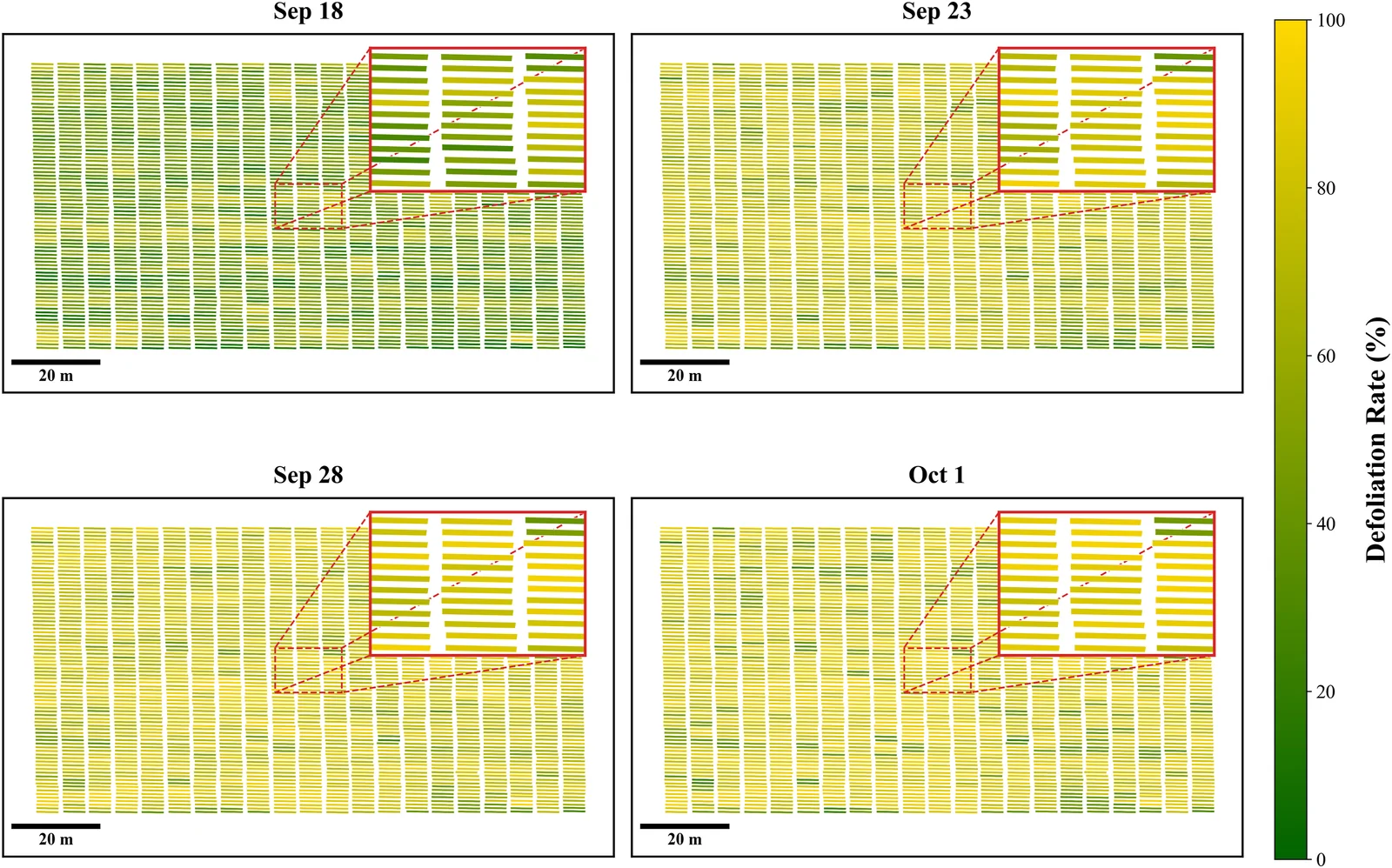
Spatial distribution of predicted cotton defoliation rates at 5, 10, 15, and 18 days after treatment (DAT). Each elongated rectangle represents an individual plot assigned its predicted defoliation rate. Insets highlight representative areas showing contrasting temporal patterns. Warmer colors indicate higher predicted defoliation rates.

## 4 Discussion

### 4.1 Spectral indicators provide more informative predictors of post-treatment defoliation

Accurate monitoring of cotton defoliation is important for optimizing harvest schedules and improving mechanical harvesting efficiency. Remote sensing approaches commonly use spectral and textural features to assess crop physiological and structural conditions [30], [31]. In this study, spectral indices consistently showed stronger relationships with defoliation rate (DR) and higher predictive performance than texture features, indicating that spectral information was more responsive to post-treatment canopy changes under the conditions examined.

This difference may be related to known physiological and structural changes during chemical defoliation, although these intermediate traits were not independently measured in the present study. Prior to widespread leaf abscission, chemical defoliants (e.g., thidiazuron, and ethephon) induce biochemical senescence, which consequently depletes chlorophyll and alters the optical signatures of the leaves [32]. Vegetation indices derived from the near-infrared (NIR) and red-edge wavelengths are sensitive to variations in canopy pigment status and moisture content [33], enabling early detection of senescence-related changes. In contrast, GLCM-based texture features primarily characterize spatial heterogeneity and canopy roughness [21], [34]. Their relatively weak relationships with DR may reflect the fact that structural changes become more pronounced only after substantial leaf loss and may also be affected by canopy gaps, shadows, and increasing soil exposure.

Combining spectral and texture features did not improve prediction relative to spectral indices alone, suggesting that the texture variables provided limited complementary information in the present dataset. More generally, increasing feature dimensionality does not necessarily improve prediction when additional variables contain redundant or noisy information. These findings highlight the importance of selecting informative predictors rather than simply combining multiple feature types in UAV-based defoliation monitoring.

### 4.2 Temporal information provides complementary information for dynamic defoliation modeling

Cotton defoliation is a progressive physiological process, and temporal information can therefore contribute to the characterization of changes that cannot be fully represented by single-date observations [4], [35]). In this study, adding days after treatment (DAT) to the relative spectral-change features increased RF performance from an R^2^ of 0.71 to 0.72 and reduced RMSE from 11.07% to 10.94%. Although the improvement was modest, it indicates that elapsed time after treatment provided complementary information beyond the UAV-derived spectral changes.

Unlike categorical phenological stages, continuous DAT provides a direct temporal reference that may help distinguish similar spectral states occurring at different phases of post-treatment progression [36]. This may be particularly useful in diverse germplasm populations, where accessions can exhibit comparable canopy reflectance at a given time while differing in the subsequent rate of defoliation. Nevertheless, the limited improvement after adding DAT indicates that most predictive information was already contained in the relative spectral-change features. Temporal encoding should therefore be regarded as a complementary rather than dominant component of the model.

The model comparison also showed that RF outperformed the evaluated deep-learning architectures under the present dataset. Although LSTM and related networks are well suited to modeling nonlinear temporal relationships [37], their performance depends strongly on the amount of independent training information available. Here, the ground-truth dataset comprised only 48 unique accessions, which may have limited the generalization of higher-capacity models. In contrast, RF showed better predictive performance under accession-grouped validation. These results emphasize the importance of matching model complexity to the effective sample size when modeling dynamic crop traits.

### 4.3 Feature optimization reduces redundancy while maintaining predictive performance

Multispectral datasets often contain substantial redundancy because many vegetation indices are derived from overlapping spectral bands. In this study, correlation analysis confirmed strong collinearity among spectral predictors. Such redundancy can increase model complexity and instability without necessarily improving generalization [38].

The EWM framework integrated RF importance, mutual information, PCA loadings, and Pearson and Spearman correlations to rank candidate features. RF importance received the largest weight (68.2%), indicating that model-based nonlinear information contributed substantially to the integrated ranking. However, the difference in predictive performance between the EWM-based and equal-weight rankings was minimal (R^2^ = 0.682 vs. 0.681), suggesting that the main value of feature optimization was not a large gain in accuracy but rather the reduction of redundant predictors while maintaining predictive performance. This interpretation is consistent with previous studies emphasizing the importance of feature selection in reducing redundancy and improving model efficiency.

### 4.4 Phenotypic variation in post-treatment defoliation trajectories

The BLUP-based trajectory analysis revealed substantial variation in post-treatment defoliation among cotton accessions. PCA showed that PC1 explained 87.16% of the total variance, with all observation dates contributing similarly, indicating that accession-level differences were driven mainly by the overall magnitude and rate of defoliation rather than fundamentally different temporal patterns.

The four trajectory groups provided a useful summary of this variation. Rapid-response accessions maintained higher defoliation rates, whereas slow-response accessions showed more gradual progression, with the remaining groups exhibiting intermediate patterns. Such differences are agronomically relevant because the timing and uniformity of defoliation directly influence harvest scheduling and mechanical picking efficiency. Compared with single end-point measurements, trajectory-based phenotyping provides a more complete description of dynamic crop responses [39].

Differences among the retrospectively defined groups were visible at the first post-treatment observation and became more pronounced over time. These trajectories therefore provide a practical basis for comparing germplasm under common management conditions and identifying materials with favorable defoliation characteristics for mechanized harvesting.

Although the present study was conducted at a single location during one growing season, the proposed framework provides a useful basis for broader multi-environment validation. Future studies integrating dynamic UAV phenotypes with genomic information may further clarify the genetic basis of defoliation dynamics and enhance their value for cotton breeding.

## 5 Conclusion

This study developed a multi-temporal UAV-based machine-learning framework for characterizing post-treatment cotton defoliation dynamics across diverse germplasm. First, spectral indices provided more informative predictors than texture features, and relative changes from the pre-treatment baseline further improved defoliation-rate estimation. Second, temporal information provided a modest but complementary contribution to model performance, with the optimal Random Forest model achieving an R^2^ of 0.73 and an RMSE of 10.69%. Third, accession-level trajectory analysis identified four post-treatment defoliation patterns, with variation mainly reflected in the magnitude and rate of defoliation progression. Overall, the proposed framework provides a scalable and non-destructive approach for high-throughput characterization of cotton defoliation dynamics and may support germplasm evaluation and mechanized harvest management.

## Author contributions

Mingze Li: Writing—Original Draft, Data Curation, Software, Validation, Methodology, Formal Analysis. Qisheng Feng: Writing—Review and Editing, Validation, Data Management. Xiaofei Li, Guoping Wang, Mingwei Du, Sumei Wan, Hongqiang Dong: Data Management, Formal Analysis, Data Curation, Conceptualization. Mengyan Ren: Data Collection, Validation. Zuoren Yang: Resources. Guodong Chen: Validation, Resources. Lu Feng: Supervision, Resources, Funding Acquisition, Revision and Editing.

## Data availability statement

The full reproducible Python code for ten regression models of cotton defoliation rate prediction, alongside the PCA clustering analysis scripts, sample test Excel datasets from the cotton variety trial, and trained model weights are deposited in the open GitHub repository: https://github.com/LiMingZe-1222/Ten-regression-models-for-defoliation-rate-prediction-.git. The complete raw field experimental datasets are large and can be made available from the corresponding author upon reasonable request for non-commercial research purposes. Detailed step-by-step running instructions are documented in the repository README file for model testing and result reproduction.

## Declaration of competing interest

The authors declare that they have no known competing financial interests or personal relationships that could have appeared to influence the work reported in this paper.
