# Supplementary material for: Multi-temporal UAV multispectral phenotyping and machine learning for modeling cotton defoliation dynamics across diverse accessions
Source: Plant Phenomics. 2026 Oct 13;8(4):100295. doi: 10.1016/j.plaphe.2026.100295 (PMC13718646; doi:10.1016/j.plaphe.2026.100295)
Supplement: Supplementary file 1 [file mmc1.docx]

**Fig. A.1** Determination of the optimal cluster number (k). Variation of the sum of squared errors (SSE), silhouette coefficient, Calinski–Harabasz index, and Davies–Bouldin index with increasing cluster number. The dashed line indicates the selected value (k = 4).

Fig. A.1

**Table A.1** Summary of regression models, hyperparameter configurations, and optimization strategies for cotton defoliation estimation.

| Model | key hyperparameters |
| --- | --- |
| LSTM | feature attention mechanism, number of hidden layer nodes (32), number of layers (1), dropout rate (0.3), epochs (150), optimizer (Adam, learning rate=0.005, weight_decay=1e-3, learning rate scheduler incorporated) |
| CNN | number of convolutional filters (16/32), kernel size (3), number of fully connected layer nodes (64), learning rate (0.001), optimizer (Adam) |
| DNN | number of hidden layer nodes (128/64/32), dropout rate (0.2), learning rate (0.001), optimizer (Adam) |
| SVM | kernel function (RBF), inner cross-validation folds for hyperparameter tuning (3-fold), grid search parameter ranges (C: [0.1, 1, 10, 100, 500]; gamma: ['scale', 'auto', 0.1, 0.01]; epsilon: [0.001, 0.01, 0.05, 0.1]) |
| RF | number of decision trees (100) |
| Ridge | regularization coefficient α |
| MLP | number of hidden layer nodes (100/50), maximum number of iterations (500) |
| GBDT | number of estimators (100), learning rate (0.1) |
| XGBoost | number of estimators (100), learning rate (0.1) |
| Stacking | base models (SVM, RF, GBDT, XGBoost), meta-model (Ridge, α=1.0), internal out-of-fold prediction strategy (5-fold KFold) |

**Table A.2** Top 25 optimal features

| Features | Pearson | Spearman | RandomForest | MutualInfo | PCA | Composite_Score |
| --- | --- | --- | --- | --- | --- | --- |
| RARI | 0.80 | 0.75 | 0.52 | 0.54 | 0.17 | 0.98 |
| NDVI | 0.77 | 0.71 | 0.10 | 0.46 | 0.14 | 0.39 |
| OSAVI | 0.62 | 0.68 | 0.03 | 0.46 | 0.22 | 0.32 |
| NDREI | 0.74 | 0.74 | 0.04 | 0.44 | 0.19 | 0.32 |
| EVI2 | 0.53 | 0.67 | 0.03 | 0.45 | 0.22 | 0.30 |
| MSR | 0.76 | 0.68 | 0.02 | 0.40 | 0.15 | 0.27 |
| SAVI | 0.49 | 0.65 | 0.01 | 0.44 | 0.22 | 0.27 |
| SAVI2 | 0.49 | 0.65 | 0.01 | 0.44 | 0.22 | 0.27 |
| IPVI | 0.75 | 0.69 | 0.01 | 0.43 | 0.14 | 0.26 |
| MSARI | 0.65 | 0.55 | 0.01 | 0.28 | 0.22 | 0.24 |
| GNDVI | 0.71 | 0.69 | 0.01 | 0.35 | 0.14 | 0.24 |
| CARI | 0.60 | 0.55 | 0.01 | 0.28 | 0.23 | 0.23 |
| SR | 0.72 | 0.63 | 0.01 | 0.31 | 0.15 | 0.23 |
| NGI | 0.62 | 0.64 | 0.01 | 0.37 | 0.14 | 0.23 |
| RVI | 0.72 | 0.63 | 0.01 | 0.31 | 0.15 | 0.23 |
| MCARI | 0.52 | 0.49 | 0.00 | 0.26 | 0.23 | 0.21 |
| RDVI | 0.46 | 0.49 | 0.01 | 0.28 | 0.23 | 0.21 |
| TVI | 0.46 | 0.44 | 0.00 | 0.20 | 0.22 | 0.19 |
| DVI | 0.43 | 0.41 | 0.00 | 0.17 | 0.22 | 0.17 |
| RESAVI | 0.31 | 0.36 | 0.01 | 0.23 | 0.18 | 0.17 |
| Spray defoliant | 0.46 | 0.49 | 0.01 | 0.14 | 0.19 | 0.17 |
| GI | 0.49 | 0.39 | 0.01 | 0.19 | 0.11 | 0.15 |
| NIR | 0.34 | 0.32 | 0.00 | 0.09 | 0.21 | 0.14 |
| RedEdge | 0.33 | 0.31 | 0.00 | 0.08 | 0.21 | 0.13 |
| WDRVI2 | 0.11 | 0.42 | 0.02 | 0.32 | 0.04 | 0.12 |

**Table A.3** Principal component loadings of the four observation dates on PC1 and PC2

| **Observation_Period** | **PC1_Loading** | **PC2_Loading** |
| --- | --- | --- |
| Sep.18 | 0.45 | 0.88 |
| Sep.23 | 0.52 | -0.19 |
| Sep.28 | 0.52 | -0.21 |
| Oct.1 | 0.51 | -0.38 |
